# Analysis of Mechanisms for Electron Uptake by *Methanothrix harundinacea* 6Ac During Direct Interspecies Electron Transfer

**DOI:** 10.3390/ijms26094195

**Published:** 2025-04-28

**Authors:** Lei Wang, Xiaoman Shan, Yanhui Xu, Quan Xi, Haiming Jiang, Xia Li

**Affiliations:** 1 School of Life Science and Technology, Inner Mongolia University of Science and Technology, Baotou 014010, China; mmisspark@imust.edu.cn (L.W.); sxm2000@stu.imust.edu.cn (X.S.); xvyh@stu.imust.edu.cn (Y.X.); 2 School of Mining and Coal, Inner Mongolia University of Science and Technology, Baotou 014010, China; xiquan99@stu.imust.edu.cn

**Keywords:** direct interspecies electron transfer, reduction of carbon dioxide to methane, *Methanothrix harundinacea* 6Ac, electron and proton flux, proposed model

## Abstract

Direct interspecies electron transfer (DIET) is a syntrophic metabolism wherein free electrons are directly transferred between microorganisms without the mediation of intermediates such as molecular hydrogen or formate. Previous research has demonstrated that *Methanothrix harundinacea* 6Ac is capable of reducing carbon dioxide through DIET. However, the mechanisms underlying electron uptake in *M. harundinacea* 6Ac during DIET remain poorly understood. This study aims to elucidate the electron and proton flux in *M. harundinacea* 6Ac during DIET and to propose a model for electron uptake in this organism, primarily based on the analysis of gene transcript levels, genomic characteristics of *M. harundinacea* 6Ac, and the pathways generating fully reduced ferridoxin (Fd_red_^2−^), reduced coenzyme F_420_ (F_420_H_2_), coenzyme M (CoM-SH), and coenzyme B (CoB-SH) during DIET. The findings suggest that membrane-bound heterodisulfide reductase (HdrED), F_420_H_2_-dehydrogenase lacking subunit F (Fpo^−^), and cytoplasmic heterodisulfide reductase (HdrABC)-subunit B of F_420_-reducing hydrogenase (FrhB) complex play critical roles in electron uptake in *M. harundinacea* 6Ac during DIET. Specifically, Fpo^−^ is responsible for generating Fd_red_^2−^ with reduced methanophenazine (MPH_2_), driven by a proton motive force, while HdrED facilitates the reduction of heterodisulfide of coenzyme M and coenzyme B (CoM-S-S-CoB) to CoM-SH and CoB-SH using MPH_2_. Additionally, cytoplasmic heterodisulfide reductase HdrABC and subunit B of coenzyme F_420_-hydrogenase complex (HdrABC-FrhB complex) catalyzes the reduction of oxidized coenzyme F_420_ (F_420_) to F_420_H_2_, utilizing CoM-SH, CoB-SH, and Fd_red_^2−^. This study represents the first genetics-based functional characterization of electron and proton flux in *M. harundinacea* 6Ac during DIET, providing a model for further investigation of electron uptake in *Methanosaeta* species. Furthermore, it deepens our understanding of the mechanisms underlying electron uptake in methanogens during DIET.

## 1. Introduction

For years, interspecies H_2_ and formate transfer were regarded as the dominant mechanisms for microbial electron exchange [1,2,3]. During syntrophic interspecies hydrogen (H_2_) and formate transfer, acetogens metabolize primary fermentation products (e.g., propionate, butyrate, and ethanol) to produce acetate, formate, and H_2_. These metabolic byproducts are subsequently utilized by partner microorganisms, including methanogens [3,4,5], sulfate-reducing bacteria [6], or nitrate/nitrite-reducing bacteria [7,8,9,10]. The efficiency of this syntrophic degradation of fermentation intermediates is critically dependent on maintaining low H_2_ concentrations, as this thermodynamic constraint ensures the favorable directionality of the reaction. Such interspecies H_2_ and formate transfer mechanisms have been extensively documented in diverse anaerobic co-culture systems [11,12,13,14,15].

**Figure 1:**
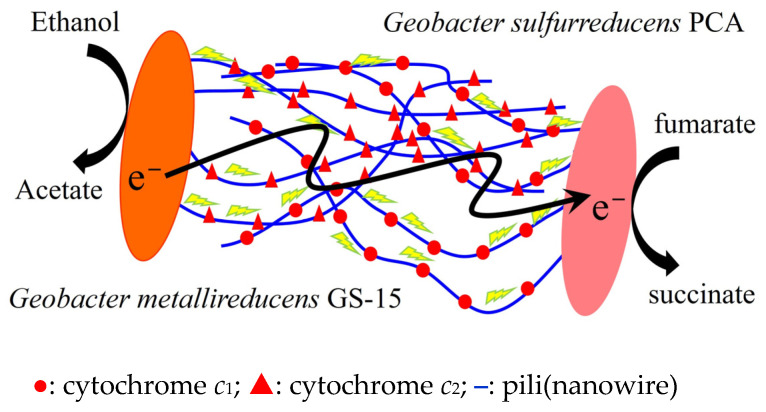
Model for direct interspecies electron transfer between *G. metallireducens* GS-15 and *G. sulfurreducens* PCA adapted from Lovley [26].

However, emerging evidence suggests that direct interspecies electron transfer (DIET) may serve as a viable alternative to indirect or mediated interspecies electron transfer pathways. DIET enables direct electron flow between microbial species through bioelectrical connections or conductive materials, bypassing the need for soluble electron shuttles. This phenomenon was first demonstrated in a defined co-culture system where *Geobacter metallireducens* GS-15 and *Geobacter sulfurreducens* PCA collaboratively reduced fumarate to succinate using ethanol as an electron donor substrate (Figure 1) [16]. Subsequent studies have identified numerous pure co-cultures capable of DIET, with most documented cases involving syntrophic partnerships between *Geobacter* species and methanogenic archaea [17,18,19,20,21,22,23,24,25].

Since the discovery of DIET, significant research efforts have focused on elucidating its key components, including: (i) electron-donating microorganisms [22,24,25,27,28,29,30], (ii) electron-accepting microorganisms [17,18,19,21,22,23,30,31,32,33], (iii) the extracellular electron transfer mechanisms employed by electron-donating microorganisms [23,34], and (iv) the extracellular electron uptake processes of electron-accepting microorganisms [23,28,31,35,36], and effect of (semi)conductive material on DIET.

While previous studies have demonstrated that certain microorganisms can directly accept electrons from syntrophic partners through direct cell contact or via (semi)conductive materials, the molecular mechanisms governing extracellular electron uptake remain poorly understood. To address this knowledge gap, transcriptomic analysis was employed to elucidate the key enzymes and proteins involving in extracellular electron uptake, along with electron and proton flux during CO_2_ reduction to methane in *Methanosarcina barkeri* 800 [28,35,36], *Methanosarcina acetivorans* strain WWM1(Δ*hpt*) [31], and *Methanothrix thermoacetophila* [23] during DIET.

**Figure 2:**
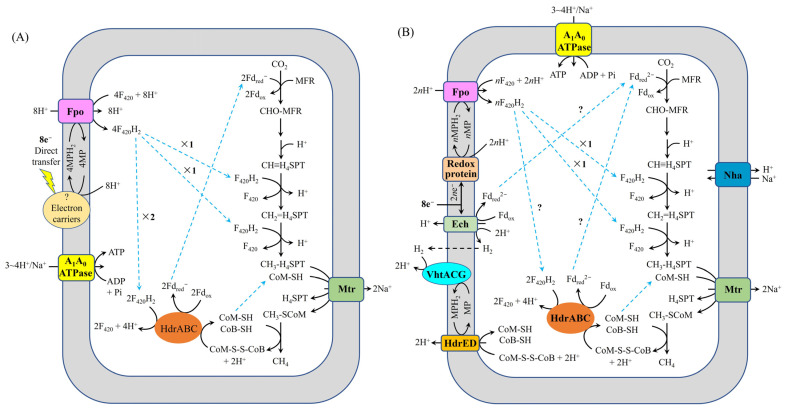
Proposed model for electron and proton flux for carbon dioxide reduction to methane in *M. barkeri* 800 during DIET-based growth. (**A**) adapted from Holmes et al. [35]. (**B**) adapted from Huang et al. [28]. H_4_MPT, tetrahydromethanopterin; MFR, methanofuran; Mtr, Na^+^-translocating methyl-H_4_MPT:coenzyme M methyltransferase; CoM-S-S-CoB, heterodisulfide of coenzyme M and coenzyme B; CoB-SH, coenzyme B; CoM-SH, coenzyme M; Fd_red_^−^, one-electron reduced ferredoxin; Fd_red_^2−^, fully reduced ferredoxin; Fd_ox_, oxidized ferredoxin; F_420_, oxidized coenzyme F_420_; F_420_H_2_, reduced coenzyme F_420_; MP, oxidized methanophenazine; MPH_2_, reduced methanophenazine; HdrABC, cytoplasmic heterodisulfide reductase; Mcr, methyl-coenzyme M reductase; Fpo, F_420_H_2_ dehydrogenase; Ech, ferredoxin-dependent hydrogenase; VhtACG, methanophenazine-reducing hydrogenases; A_1_A_0_ATPase, ATP synthase; Nha, Na^+^/H^+^ antiporter; *n*, the amount of F_420_H_2_ generated by Fpo (2 < *n* < 3); “?”, it indicates uncertainty.

*M. barkeri* 800 represents the first microorganism for which the molecular mechanism underlying the reduction of carbon dioxide to methane via DIET has been comprehensively elucidated. Holmes et al. [35] proposed a metabolic model for CO₂-reductive methanogenesis in *M. barkeri* 800 during DIET by conducting comparative transcriptomic analysis between two syntrophic growth modes (Figure 2A): (i) DIET-based coculture with *Geobacter metallireducens* GS-15 and (ii) H_2_-mediated interspecies transfer (HIT) with *Pelobacter carbinolicus*. Their transcriptomic data revealed that F_420_H_2_ dehydrogenase (Fpo) and cytoplasmic heterodisulfide reductase HdrABC (HdrABC) were significantly upregulated during DIET, suggesting their crucial involvement in extracellular electron uptake in *M. barkeri* 800 during DIET. Fpo catalyzes the reduction of oxidized coenzyme F_420_ (F_420_) to reduced coenzyme F_420_ (F_420_H_2_) using reduced methanophenazine (MPH_2_) driven by proton motive force, while HdrABC utilizes a portion of the generated F_420_H_2_ to simultaneously reduce both heterodisulfide of coenzyme M and coenzyme B (CoM-S-S-CoB) and oxidized ferredoxin (Fd_ox_), yielding coenzyme M (CoM-SH), coenzyme B (CoB-SH), and one-electron reduced ferredoxin (Fd_red_^−^), respectively. The proposed model suggests that the membrane-bound heterodisulfide reductase HdrED was not involved in CoM-S-S-CoB reduction in *M. barkeri* 800 during DIET. Huang et al. [28] established an alternative model for CO_2_-reductive methanogenesis in *M. barkeri* 800 during DIET with *Rhodopseudomonas palustris*, based on transcriptomic profiling of DIET-grown cocultures (Figure 2B). Their analysis revealed significant upregulation of genes encoding key respiratory complexes, including: the energy-converting hydrogenase (Ech), the membrane-bound F_420_H_2_ dehydrogenase (Fpo), the methanophenazine-reducing hydrogenase (VhtACG), and both heterodisulfide reductase systems (HdrED and HdrABC). These findings suggest that these enzyme complexes collectively facilitate extracellular electron uptake during DIET in *M. barkeri* 800. Notably, the model proposed by Holmes et al. [35] suggests that HdrED does not participate in either CoM-S-S-CoB reduction or extracellular electron transfer, contrasting with Huang et al.′s findings [28]. In addition, under physiological conditions (pH 7.0; F_420_/F_420_H_2_ ratio ≤ 0.1; 37 °C), thermodynamic analysis reveals that the free energy change (Δ*G*′) for F_420_ reduction by MPH_2_ coupled to proton motive force ranges from + 0.41 to +13.91 kJ/mol, while CoM-S-S-CoB reduction by MPH_2_ exhibits a more favorable Δ*G*′ range of −9.55 to + 3.95 kJ/mol. This significant energetic difference indicates that CoM-S-S-CoB reduction is thermodynamically preferred over F_420_ reduction under these conditions. Consequently, these calculations support the hypothesis that in *M. barkeri* 800 during DIET, HdrED likely utilizes MPH_2_ as the immediate electron donor for CoM-S-S-CoB reduction [28]. These apparent discrepancy highlights the need for additional mechanistic studies. Furthermore, Wang et al. [36] identified a functional role for the F_420_-reducing hydrogenase (FrhABG) in electron transfer processes in *M. barkeri* 800 during DIET. The finding suggests that FrhABG catalyzes the oxidation of a portion of the generated F_420_H_2_ to produce H_2_, consistent with the observed upregulation of *frh*ABG gene expression under DIET conditions. These findings collectively demonstrate that in Type I *Methanosarcina* species (e.g., *M. barkeri* 800), both the extracellular electron uptake mechanisms and intracellular electron transfer pathways exhibit greater complexity than previously anticipated, warranting further investigation.

**Figure 3:**
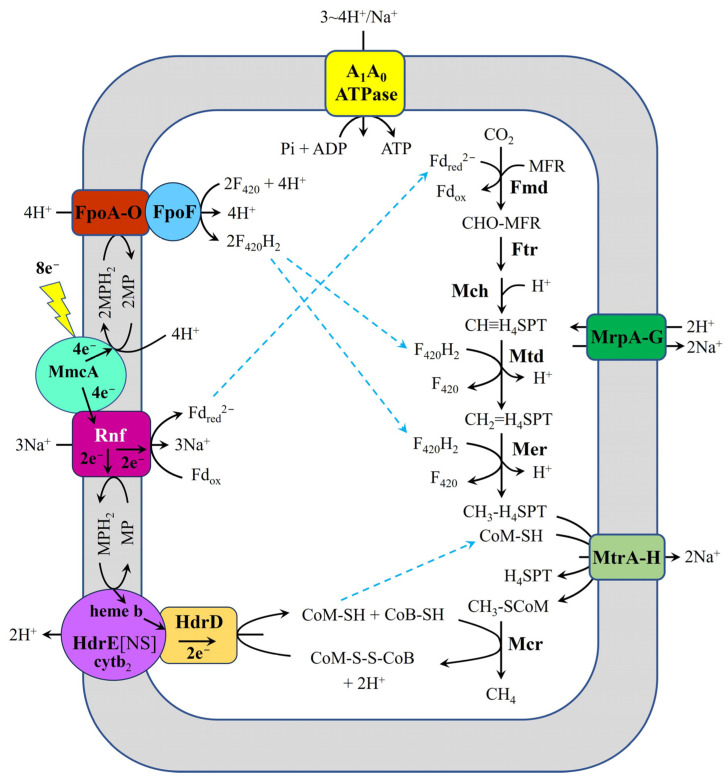
Proposed model for electron and proton flux in *M. acetivorans* strain WWM1(Δ*hpt*) during DIET with *G. metallireducens* GS-15, adapted from Holmes et al. [31]. Ethanol is provided as the source of electrons. H_4_MPT, tetrahydromethanopterin; MFR, methanofuran; Mtr, Na^+^-translocating methyl-H_4_MPT:coenzyme M methyltransferase; CoM-S-S-CoB, heterodisulfide of coenzyme M and coenzyme B; CoB-SH, coenzyme B; CoM-SH, coenzyme M; Fd_red_^2−^, fully reduced ferredoxin; Fd_ox_, oxidized ferredoxin; F_420_, oxidized coenzyme F_420_; F_420_H_2_, reduced coenzyme F_420_; MP, oxidized methanophenazine; MPH_2_, reduced methanophenazine; HdrED, heterodisulfide reductase; Mcr, methyl-coenzyme M reductase; Fpo, F_420_H_2_ dehydrogenase; A_1_A_0_ATPase, ATP synthase; MmcA, outer-surface multiheme *c*-type cytochrome; MrpA-G, H^+^/Na^+^ antiporter complex; Rnf, Na^+^-translocating ferredoxin: methanophenazine reductase complex.

*Methanosarcina acetivorans* strain WWM1(Δ*hpt*) represents another *Methanosarcina* species for which potential extracellular electron uptake mechanisms during DIET with *G. metallireducens* GS-15 have been investigated. Holmes et al. [31] systematically characterized the electron and proton transfer pathways in *M. acetivorans* WWM1(Δ*hpt*) during DIET-based growth with *G. metallireducens* GS-15 (using ethanol as the sole electron donor) through integrated comparative transcriptomic approaches (Figure 3). Their analysis revealed significant upregulation of key respiratory components in DIET-grown cocultures compared to acetate-grown controls, including: (i) the outer surface multiheme *c*-type cytochrome MmcA, (ii) the Na^+^-translocating ferredoxin: methanophenazine reductase complex (Rnf complex), (iii) Fpo, and (iv) HdrED. These findings strongly suggest that these molecular components collectively mediate extracellular electron uptake in *M. acetivorans* WWM1(Δ*hpt*). Notably, the proposed electron and proton transfer pathway in this strain exhibits fundamental mechanistic differences from the system previously characterized in *M. barkeri* 800 [28,35], highlighting distinct DIET adaptation strategies among methanogens.

**Figure 4:**
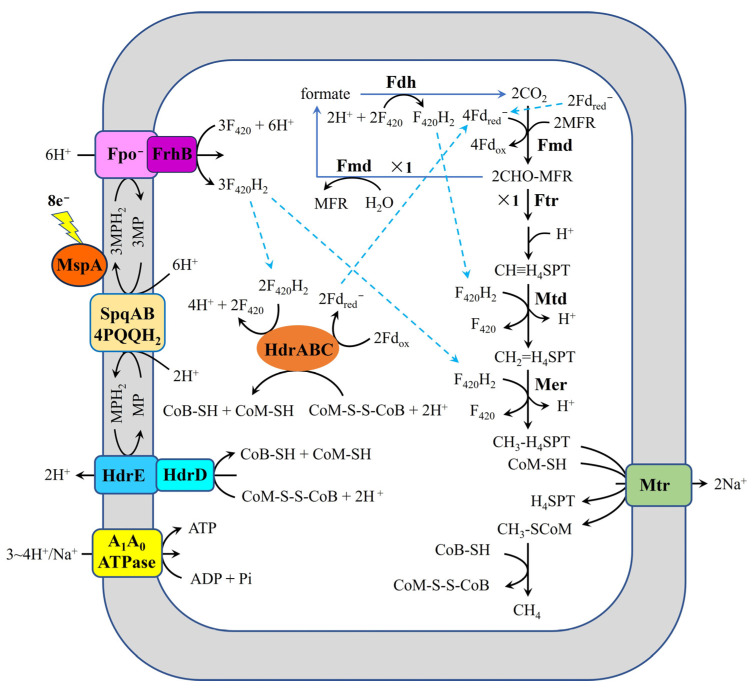
Proposed pathway for carbon dioxide reduction in *M. thermoacetophila* during DIET-based growth, adapted from Zhou et al. [23]. MspA, main sheath fiber protein; SpqAB, surface quinoprotein complex; PQQH_2_, reduced pyrroloquinoline quinone;H_4_MPT, tetrahydromethanopterin; MFR, methanofuran; Mtr, Na^+^-translocating methyl-H_4_MPT:coenzyme M methyltransferase; CoM-S-S-CoB, heterodisulfide of coenzyme M and coenzyme B; CoB-SH, coenzyme B; CoM-SH, coenzyme M; Fd_red_^−^, one electron reduced ferredoxin; Fd_ox_, oxidized ferredoxin; F_420_, oxidized coenzyme F_420_; F_420_H_2_, reduced coenzyme F_420_; MP, oxidized methanophenazine; MPH_2_, reduced methanophenazine; HdrABC, heterodisulfide reductase subunits A, B, C; HdrED, heterodisulfide reductase subunits E and D; FrhB, subunit B of F_420_-reducing hydrogenase; Mcr, methyl-coenzyme M reductase; Fpo^−^, F_420_H_2_ dehydrogenase lacking subunit F; A_1_A_0_ATPase, ATP synthase; Fdh, formate-dehydrogenase complex; Fmd, formylmethanofuran dehydrogenase complex.

Recent work by Zhou et al. [23] has elucidated a novel mechanism for DIET-mediated methanogenesis in *Methanothrix thermoacetophila* through comparative transcriptomic analysis of DIET-grown cocultures (with *G. metallireducens* GS-15) versus acetoclastic growth conditions (Figure 4). The study revealed significant upregulation of genes encoding several key components: (i) the surface-associated primary sheath fiber protein (MspA), (ii) the surface quinoprotein complex (SqpAB), (iii) heterodisulfide reductases (HdrED and HdrABC), (iv) formylmethanofuran dehydrogenase (Fmd), (v) formate dehydrogenase (Fdh), (vi) Fpo^−^, and (vii) subunit B of FrhABG (FrhB). These findings suggest that this unique ensemble of surface proteins and redox enzymes collectively facilitates extracellular electron uptake in *M. thermoacetophila*, representing a distinct DIET adaptation mechanism among methanogens. This proposed model postulates that partial F_420_H_2_ production requires coupling of Fmd and Fdh with two moles of one-electron reduced ferredoxin (Fd_red_^−^) generated from acetate oxidation in *M. thermoacetophila*. However, this hypothesis warrants critical examination, as several methanogens—including *Methanosarcina harundinacea* 6Ac [37] and *M. barkeri* 800 [36]—demonstrate the capability for DIET-based CO_2_ reduction to methane in the absence of acetate. This apparent discrepancy could be resolved experimentally by determining whether *G. metallireducens* GS-15/*M. thermoacetophila* cocultures can couple methane production with the oxidation of alternative electron donors such as propanol or butanol. The proposed mechanism whereby the Fpo^−^-FrhB complex mediates F_420_ reduction to F_420_H_2_ using MPH_2_ driven by proton motive force also requires careful consideration. This hypothesis is challenged by experimental evidence demonstrating that a subunit F of Fpo (FpoF)-deficient strain of *Methanosarcina mazei* Gö1, despite retaining both Fpo^−^ and FrhB components, was incapable of direct F_420_H_2_ oxidation via membrane-bound electron transport [38]. These findings not only question the functional capacity of Fpo^−^-FrhB in F_420_H_2_ generation but also necessitate re-examination of the proposed coupled system where HdrABC generates Fd_red_^−^ through F_420_H_2_ oxidation. These findings collectively highlight the need for more comprehensive investigations into both the extracellular electron uptake mechanisms and intracellular electron transfer pathways during DIET-mediated methanogenesis in *M. thermoacetophila*.

Although several unresolved questions remain regarding extracellular electron uptake mechanisms and intracellular electron transfer pathways during DIET-mediated methanogenesis in the studied methanogens, these findings nevertheless provide valuable insights for investigating similar processes in other methanogenic species. Furthermore, these findings motivate and direct further research in this emerging field. *Methanothrix harundinacea* 6Ac (formerly *named Methanosaeta harundinacea* 6Ac) (JCM-13211, https://jcm.brc.riken.jp/) has also been reported to perform DIET-dependent methanogenesis [19,37], however, the extracellular electron uptake and intracellular electron transfer pathways in this archaeon remains unresolved. This study aims to elucidate these mechanisms by: (i) analyzing transcriptomic profiles of *M. harundinacea* 6Ac during DIET-based growth, (ii) examining genomic features associated with electron uptake, and (iii) determining the pathways for generating fully reduced ferredoxin (Fd_red_^2−^), F_420_H_2_, CoM-SH, and CoB-SH. Based on these analyses, we propose a model for extracellular electron uptake in *M. harundinacea* 6Ac via DIET. This model will serve as a foundational framework for future investigations into electron uptake mechanisms in *Methanosaeta* spp. and broaden our understanding of DIET-based methanogenesis in anaerobic microbial communities.

## 2. Possible Mechanisms for Extracellular Electron Uptake by *M. harundinacea* 6Ac

As a member of the *Methanosarcinales*, *M. harundinacea* 6Ac possesses cytochromes [39], which are typically localized between the cytoplasmic membrane and S-layer in this order [40]. Genomic analysis reveals that *M. harundinacea* 6Ac encodes key redox-active components, including a class I c-type cytochrome (Mhar_1883), cytochrome *c* biogenesis proteins (Mhar_0020, Mhar_1541, Mhar_1552), and multiple S-layer proteins (Mhar_0562, Mhar_0637, Mhar_0867, Mhar_1021, Mhar_1810) [19]. Notably, the gene encoding the class I cytochrome (Mhar_1883) was highly expressed during DIET-based syntrophic growth with *G. metallireducens* GS-15, where ethanol was stoichiometrically converted to methane [19]. Given that *c*-type cytochromes in *Methanosarcina* species exhibit midpoint potentials of −230 ± 10 mV and −140 ± 10 mV (vs. SHE) [41], we propose a thermodynamically feasible electron transfer pathway in *M. harundinacea* 6Ac: Electrons may flow from cytochrome *c* (−230 ± 10 mV) to oxidized methanophenazine (MP) (*E°*′ = −165 ± 6 mV [42]), yielding a favorable Gibbs free energy change (Δ*G*′ = −9.33 kJ/mol at 37 °C, pH 7, assuming equal substrate and product concentrations). Collectively, these findings support a model where *c*-type cytochromes in *M. harundinacea* 6Ac may directly accept extracellular electrons and transfer them to MP, generating MPH_2_ to drive methanogenesis. This mechanism appears analogous to that observed in *M. acetivorans* strain WWM1(Δ*hpt*), where the outer surface multiheme *c*-type cytochrome MmcA was proposed to mediate direct electron uptake from syntrophic partners during DIET [31]. Nevertheless, to comprehensively establish the mechanistic details of these proposed electron transfer pathways, molecular-level validation is essential.

## 3. Possible Mechanisms for Generation of Fd_red_^2−^ in *M. harundinacea* 6Ac During DIET

**Figure 5:**
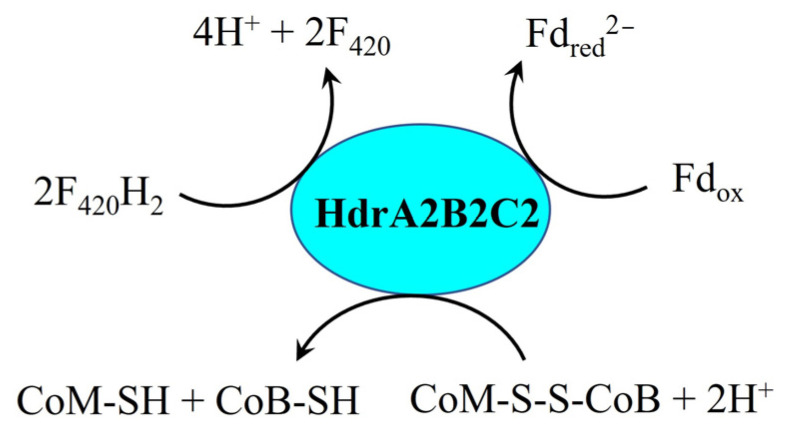
Electron transfer reactions catalyzed by HdrA2B2C2 [47,48]. HdrA2B2C2, cytoplasmic heterodisulfide reductase subunits A2, B2, C2; CoM-S-S-CoB, heterodisulfide of coenzyme M and coenzyme B; CoB-SH, coenzyme B; CoM-SH, coenzyme M; Fd_red_^2−^, fully reduced ferredoxin; Fd_ox_, oxidized ferredoxin; F_420_, oxidized coenzyme F_420_; F_420_H_2_, reduced coenzyme F_420_.

In cytochrome-containing methanogens, membrane-bound energy conserving hydrogenase (Ech), Rnf complex, Fpo^−^, and HdrABC are typically associated with Fd_red_^2−^ oxidation or generation. However, genomic analysis reveals that *M. harundinacea* 6Ac lacks both *ech* and *rnf* genes [43], excluding their involvement in membrane-bound electron transport during DIET with *G. metallireducens* GS-15. Instead, this organism possesses a complete *fpo*^−^ gene cluster (*fpo*ABCDEHIJKLMNO) [43], which was highly expressed during DIET-based growth [19]. Notably, Fpo^−^ cannot oxidize F_420_H_2_, as demonstrated by studies with *Methanosarcina mazei* Gö1 Δ*fpo*F mutants (an FpoF-deficient strain of *M. mazei* Gö1) [38]. This demonstrates that F_420_H_2_ cannot be generated by Fpo^−^ with MPH_2_ driven by a proton motive force in *M. harundinacea* 6Ac during DIET. Consequently, it rules out the possibility of F_420_H_2_-dependent Fd_red_^2−^ generation via cytoplasmic heterodisulfide reductase HdrA2B2C2 in *M. harundinacea* 6Ac during DIET (Figure 5). Nevertheless, accumulating evidence indicates that Fpo^−^ can oxidize Fd_red_^2−^ in both *Methanosaeta* spp. [44,45] and *M. mazei* Δ*ech* mutants [46]. Cumulatively, the evidence suggests that, in *M. harundinacea* 6Ac, Fpo^−^ likely catalyzes Fd_red_^2−^ generation using MPH_2_ driven by proton motive force during DIET, which is consistent with the high expression of its *fpo*^−^ gene cluster [19]. The function of Fpo^−^ in *M. harundinacea* 6Ac during DIET could be investigated using the Fpo-specific inhibitor *p*-chloromercuriphenyl sulfonate or sodium azide [41]. Since the generation of one mole of Fd_red_^2−^ consumes three moles of protons [44,45], during DIET in *M. harundinacea* 6Ac, eight moles of protons from its syntrophic partner can only contribute to the generation of two to three moles of Fd_red_^2−^. The generation of two to three moles of Fd_red_^2−^ requires four to six moles of electrons (in the form of MPH₂), indicating that the remaining two to four moles of electrons are transferred intracellularly via another pathway. In *Methanosaeta* spp., only membrane-bound Fpo^−^ and HdrED are involved in electron transfer. Thus, it is hypothesized that HdrED is responsible for mediating the remaining extracellular electrons into the intracellular environment.

## 4. Possible Mechanisms Underlying the Generation of CoM-SH and CoB-SH in *M. harundinacea* 6Ac During DIET

In methanogens, heterodisulfide CoM-S-S-CoB reduction can occur through four distinct pathways: (i) the cytoplasmic H_2_:heterodisulfide oxidoreductase complex (MvhADG-HdrABC complex) utilizing H_2_ [39,49,50], (ii) membrane-bound HdrED with MPH_2_ [48,51], (iii) cytoplasmic heterodisulfide reductase HdrA1B1C1 with Fd_red_^2−^ [52], or (iv) cytoplasmic heterodisulfide reductase HdrA2B2C2 with F_420_H_2_ [47]. As an obligate acetoclastic methanogen [53], *M. harundinacea* 6Ac lacks the MvhADG-HdrABC complex, eliminating the first pathway. Genomic analysis reveals only *hdr*ED and *hdr*ABC clusters in *M. harundinacea* 6Ac [43], and the absence of F_420_H_2_ during initial DIET stages precludes HdrA2B2C2 involvement. While HdrA1B1C1 represents another potential candidate, neither its encoding genes [43] nor transcriptional expression [19] have been detected in *M. harundinacea* 6Ac. Notably, *hdr*ED genes (Mhar_0792-0793) were highly expressed in *M. harundinacea* 6Ac during DIET [19], suggesting HdrED mediates CoM-S-S-CoB reduction using MPH_2_. This conclusion aligns with prior reports that HdrED is responsible for transferring parts of extracellular electrons intracellularly in methanogens during DIET-based growth [23,28,31,36]. Functional validation could employ the HdrED-specific inhibitor pyridine [41]. The generation of Fd_red_^2−^ consumes only two to three moles of MPH_2_. This indicates that at least one, but no more than two, moles of MPH_2_ remain available for the reduction of CoM-S-S-CoB, which is consistent with the proposed HdrED activity.

## 5. Possible Mechanisms for F_420_H_2_ Generation in *M. harundinacea* 6Ac During DIET

**Figure 6:**
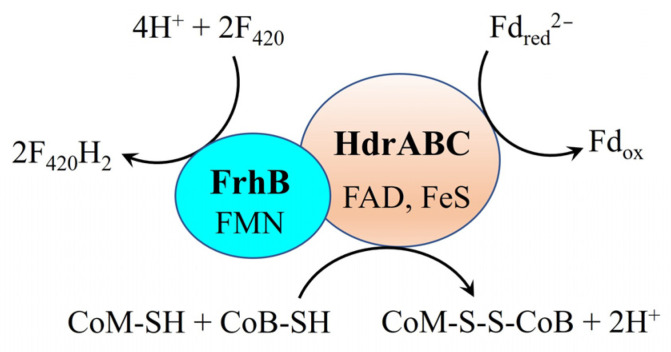
Proposed scheme for the reduction of Fd_ox_ and CoM-S-S-CoM with F_420_H_2_ through cytoplasmic HdrABC-FrhB complex [55]. Fd_red_^2−^, fully reduced ferredoxin; Fd_ox_, oxidized ferredoxin; F_420_, oxidized coenzyme F_420_; F_420_H_2_, reduced coenzyme F_420_; FrhB, subunit B of F_420_-reducing hydrogenase; CoM-S-S-CoB, heterodisulfide of coenzyme M and coenzyme B; CoM-SH, coenzyme M; CoB-SH, coenzyme B; HdrABC, heterodisulfide reductase subunits A, B, C; FAD, flavin adenine dinucleotide; FeS, iron-sulfur cluster.

F_420_H_2_, an essential electron and proton carrier for CO_2_ reduction to methane, can be produced through multiple pathways in methanogens: (i) FrhABG, fructose-inducible hydrogenase (Fru hydrogenase), or fructose-repressible hydrogenases (Frc hydrogenases) using H_2_ [39,49,54], (ii) Fdh with formate [54], (iii) HdrA1B1C1 or HdrA2B2C2 with Fd_red_^2−^ [47,52], or (iv) the HdrABC and FrhB complex (HdrABC-FrhB complex) via electron bifurcation (Figure 3) [55,56,57]. In *M. harundinacea* 6Ac, no genes for Fru/Frc hydrogenases or other subunits of FrhABG were identified, except for the gene encoding FrhB (Mhar_2358) [43]. This implies that F_420_H_2_ cannot be generated by FrhABG, Fru hydrogenase, or Frc hydrogenase during DIET in *M. harundinacea* 6Ac. As analyzed in Section 3, Fd_red_^2−^ can potentially be generated by Fpo^−^ with MPH_2_ in *M. harundinacea* 6Ac during DIET. Additionally, CoM-S-S-CoB is present in *M. harundinacea* 6Ac. Although F_420_H_2_ could potentially be produced by HdrA1B1C1 or HdrA2B2C2 with Fd_red_^2−^ and CoM-S-S-CoB, genes for HdrA1B1C1 and HdrA2B2C2 are absent from the *M. harundinacea* 6Ac genome [43], ruling out this production route. Notably, highly expressed *hdr*ABC (Mhar_0604-0607) and *frh*B (Mhar_2358) genes in *M. harundinacea* 6Ac during DIET [19] suggest the HdrABC-FrhB complex mediates F_420_H_2_ generation via electron bifurcation (Figure 6). Collectively, these findings suggest that the HdrABC-FrhB complex may facilitate the generation of F_420_H_2_ in *M. harundinacea* 6Ac during DIET. If this electron bifurcation reaction occurs during DIET in *M. harundinacea* 6Ac, it can account for the generation of F_420_H_2_ required for carbon dioxide reduction to methane. The role of the HdrABC-FrhB complex in *M. harundinacea* 6Ac during DIET-based growth can be further validated by examining the growth of the Δ*hdr*ABC/Δ*frh*B mutant of *M. harundinacea* 6Ac on DIET. If the mutant can grow on DIET, it suggests that the HdrABC-FrhB complex does not contribute to the generation of F_420_H_2_ during DIET-based growth in *M. harundinacea* 6Ac. Otherwise, it indicates the complex′s involvement in F_420_H_2_ generation during DIET-based growth.

Furthermore, an alternative pathway for partial F_420_H_2_ production involves the interaction of Fmd and Fdh utilizing Fd_red_^2−^ generated by the Fpo^−^ with MPH_2._ The significant expression of genes encoding Fmd and subunit A of Fdh in *M. harundinacea* 6Ac during DIET [19] supports this hypothesis. Moreover, this proposed pathway aligns with previous research findings [23], except for the source of Fd_red_^2−^. In the model proposed by Zhou et al. [23], the Fd_red_^2−^ utilized for partial F_420_H_2_ production through the interaction of Fmd and Fdh was derived from acetate oxidation, rather than being generated by the Fpo^−^ with MPH_2_, as discussed in this study. However, additional evidence casts doubt on the origin of Fd_red_^2−^ in this manner. For example, the co-cultures of *G. metallireducens* GS-15/*M. barkeri* 800 [36] and *G. metallireducens* GS-15/*M. harundinacea* 6Ac [37] can facilitate the syntrophic conversion of propanol and butanol to their corresponding fatty acids (propionate and butyrate) and methane via DIET. Given that *M. harundinacea* 6Ac lacks the capacity to metabolize propionate or butyrate [37,43], the Fd_red_^2−^ required for partial F_420_H_2_ production—mediated by the interaction of Fmd and Fdh—must have been generated by Fpo^−^ with MPH_2_, rather than originating from acetate oxidation. Notably, this pathway only yields limited F_420_H_2_ because the Fd_red_^2−^ consumed in this process reduces the availability of electrons for HdrED-mediated CoM-S-S-CoB reduction. This subsequently diminishes the transmembrane proton gradient, ultimately impairing ATP synthesis and negatively affecting methanogen growth. However, the necessity of this bypass route remains uncertain. Stoichiometric analyses indicate that the HdrABC-FrhB system alone can generate sufficient F_420_H_2_ to satisfy the F_420_H_2_ demand for reduction of CO_2_ to methane.

## 6. Pathway Proposed for Reduction of Carbon Dioxide in *M. harundinacea* 6Ac During DIET

**Figure 7:**
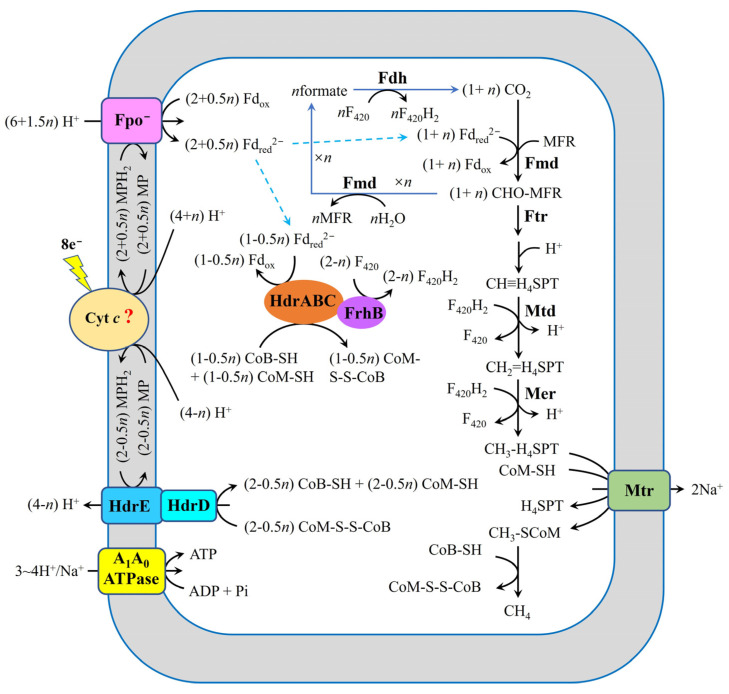
Proposed model for electron and proton flux for carbon dioxide reduction to methane in *M. harundinacea* 6Ac during DIET with *G. metallireducens* GS-15 based on the results of previous studies [19,23,38,43,44,45,53,55,56,57]. Cyt *c*, *c*-type cytochrome; H_4_MPT, tetrahydromethanopterin; MFR, methanofuran; Mtr, Na^+^-translocating methyl-H_4_MPT:coenzyme M methyltransferase; CoM-S-S-CoB, heterodisulfide of coenzyme M and coenzyme B; CoB-SH, coenzyme B; CoM-SH, coenzyme M; Fd_red_^2−^, fully reduced ferredoxin; Fd_ox_, oxidized ferredoxin; F_420_, oxidized coenzyme F_420_; F_420_H_2_, reduced coenzyme F_420_; MP, oxidized methanophenazine; MPH_2_, reduced methanophenazine; HdrABC, heterodisulfide reductase subunits A, B, C; HdrED, heterodisulfide reductase subunits E and D; FrhB, subunit B of F_420_-reducing hydrogenase; Mcr, methyl-coenzyme M reductase; Fpo^−^, F_420_H_2_ dehydrogenase (Fpo) lack of subunit F; A_1_A_0_ATPase, ATP synthase; Nha, Na^+^/H^+^ antiporter; Fdh, formate-dehydrogenase complex; Fmd, formylmethanofuran dehydrogenase complex; *n*, the amount of F_420_H_2_ generated by combining Fmd and Fdh with Fd_red_^2−^ (0 ≤ *n* < 2); “?”, it indicates uncertainty.

Building on the preceding analysis, we propose a pathway for the electron uptake in *M. harundinacea* 6Ac during growth via DIET (Figure 7). In this proposed model, eight moles of extracellular electrons are shuttled to MP through cytochrome *c*, generating four moles of MPH_2_. Subsequently, HdrED reduces a portion of CoM-S-S-CoB (more than one but less than two moles) using MPH_2_. Concurrently, Fpo^−^ reduces Fd_ox_ (more than two but less than three moles) with MPH_2_ driven by the proton motive force. Finally, F_420_H_2_ is synthesized by two distinct routes: one involving the HdrABC-FrhB complex, and the other via the combined action of Fmd and Fdh.

In *M. harundinacea* 6Ac, the mechanisms for generating reducing equivalents (MPH_2_, Fd_red_^2−^, F_420_H_2_, CoM-SH, and CoB-SH) required for CO_2_ reduction to methane during DIET differ significantly from those in traditional CO_2_-reducing methanogenesis. In cytochrome-lacking methanogens growing on CO_2_ and H_2_, Fd_red_^2−^ is produced by the energy-converting hydrogenase (Eha and/or Ehb), as well as the cytoplasmic MvhADG–HdrABC complex with H_2_. The MvhADG–HdrABC complex catalyzes the generation of CoM-SH, CoB-SH, and Fd_red_^2−^ via an electron bifurcation reaction using H_2_. F_420_H_2_ is generated by FrhABG with H_2_. In cytochrome-containing methanogens growing on CO_2_ and H_2_, Fd_red_^2−^ is produced by the Ech hydrogenase with H_2_, driven by proton motive force. The formation of CoM-SH and CoB-SH involves the interaction of VhtACG and HdrED with H_2_, while F_420_H_2_ is synthesized by FrhABG with H_2_. However, in *M. harundinacea* 6Ac during DIET-based growth, Fd_red_^2−^ is generated by the Fpo^−^ using MPH_2_ (derived from the reduction of MP with extracellular electrons) as the electron donor, driven by proton motive force. CoM-SH and CoB-SH are produced by HdrED using MPH_2_ (derived from the reduction of MP with extracellular electrons). F_420_H_2_ is generated through two distinct pathways: (i) the cytoplasmic HdrABC-FrhB complex utilizing CoM-SH, CoB-SH, and Fd_red_^2−^, and (ii) the combined activity of Fmd and Fdh with Fd_red_^2−^. This comparative analysis highlights the unique metabolic adaptations of *M. harundinacea* 6Ac for DIET-dependent methanogenesis, differing fundamentally from conventional H_2_-dependent pathways.

To better understand the biochemical mechanisms underlying extracellular electron uptake and the generation of reducing equivalents (MPH_2_, Fd_red_^2−^, F_420_H_2_, CoM-SH, and CoB-SH) in *M. harundinacea* 6Ac during DIET-based growth, we performed comparative genomic analyses of core metabolic networks across model methanogens including *M. barkeri* 800, *M. acetivorans* WWM1(Δ*hpt*), *M. thermoacetophila*, and *M. harundinacea* 6Ac (Table 1). Two critical knowledge gaps persist: (i) the molecular basis of transmembrane electron transfer in electroactive methanogens (e.g., how extracellular electrons are transferred to MP), and (ii) species-specific flux partitioning mechanisms for metabolic product reduction. Notably, both extracellular electron uptake systems and MP reduction modules exhibit significant phylogenetic divergence among methanogens.

Despite the lack of clarity regarding the initial step of methanogens accepting extracellular electrons and the mechanism by which the MP is reduced with extracellular electrons, based on the diverse energy-conservation strategies employed by methanogens, there are approximately three distinct mechanisms for the reduction of carbon dioxide to methane in methanogens undergoing DIET-dependent growth:

1. Methanogens capable of conserving energy via Fpo, such as type I *Methanosarcina* spp., including *M. bakeri* 800, may utilize the mechanism for carbon dioxide reduction to methane illustrated in Figure 2. During DIET, Fpo, HdrED, FrhABG, HdrABC, VhtACG, and Ech in these methanogens are likely to contribute to extracellular electron uptake.
2. For methanogens that conserve energy through Rnf, like type II *Methanosarcina* spp., such as *M. acetivorans* WWM1(Δ*hpt*) and *Methanosarcina horonobensis* HB-1, the mechanism for carbon dioxide reduction to methane is depicted in Figure 3. In these organisms, Fpo, Rnf, and HdrED are likely involved in extracellular electron uptake during DIET. Additionally, HdrABC may also facilitate the acquisition of extracellular electrons.
3. Methanogens that conserve energy through Fpo^−^, like *Methanothrix* spp. (formerly named *Methanosaeta* spp.), including *M. harundinacea* 6Ac and *M. thermoacetophila*, may follow the mechanism for carbon dioxide reduction to methane that is shown in Figure 7. During DIET, the Fpo^−^, HdrED, and HdrABC-FrhB complexes are potentially responsible for extracellular electron uptake in these methanogens.

Although the proposed model outlines the reduction of carbon dioxide to methane and electron uptake processes in *M. harundinacea* 6Ac during growth via DIET, the exact mechanism of electron uptake in *M. harundinacea* 6Ac during DIET remains poorly understood. Similar to all previously reported models on extracellular electron uptake in methanogens during DIET, the present proposed model has not yet been validated at the molecular level. Therefore, further experimental investigations are necessary to validate the proposed model.

## 7. Conclusions

The discovery of DIET has revealed a more efficient microbial energy-sharing mechanism than previously recognized. While significant progress has been made in DIET research, the molecular mechanisms of extracellular electron uptake in electron-accepting microbes, particularly methanogens, remain poorly understood. Here, we propose a comprehensive model for reduction of CO_2_ to methane and extracellular electron uptake in *M. harundinacea* 6Ac during DIET, integrating transcriptomic data, genomic analysis, and analysis on the mechanisms for generation of key redox components (Fd_red_^2−^, F_420_H_2_, CoM-SH, and CoB-SH). Our model identifies three critical players for electron uptake: (i) Fpo^−^ mediates Fd_red_^2−^ generation using MPH_2_ driven by proton motive force; (ii) HdrED reduces CoM-S-S-CoB to CoM-SH and CoB-SH with MPH_2_; and (ⅲ) the HdrABC-FrhB complex catalyzes F_420_ reduction to F_420_H_2_ via electron bifurcation using CoM-SH, CoB-SH, and Fd_red_^2−^.

It is worth emphasizing that, although this model is primarily constructed upon transcriptomic analyses and genomic features of *M. harundinacea* 6Ac, along with the established functions of several key enzymes (such as the Fpo^−^, HdrED, and HdrABC-FrhB complex), the molecular-level functions of the majority of enzymes engaged in electron transfer in *M. harundinacea* 6Ac during DIET-mediated growth remain uncharacterized. Future research could employ mutant strains of related enzymes or inhibitors of related enzymes to validate their functions in electron transfer during DIET. Moreover, the initial step of extracellular electron uptake by *M. harundinacea* 6Ac or how extracellular electron is transferred to MP of *M. harundinacea* 6Ac during DIET remains unclear and warrants further exploration. This exploration is essential for understanding the mechanisms of extracellular electron uptake by methanogens. This framework not only advances our understanding of *Methanothrix* spp. physiology during DIET, but it also provides new insights into DIET-based methanogenesis across diverse archaeal species.

## Figures and Tables

**Table 1:** The key differences between the multiple mechanisms of extracellular electron uptake in reported methanogens during DIET-based growth.

Microbes	Extracellular Electron Uptake	MP Reduction	F_420_ Reduction	CoM-S-S-CoB Reduction	Fd_ox_ Reduction	Reference
*M. barkeri* 800	Unknow	Unknow	Fpo with MPH_2_	HdrABC with F_420_H_2_, HdrED with MPH_2_, interaction of VhtACG and HdrED with H_2_	HdrABC with F_420_H_2_, Ech direct with extracellular electron, Ech with H_2_ from oxidation of F_420_H_2_ by FrhABG	[28,35,36]
*M. acetivorans*	MmcA	MmcA, Rnf with electrons from MmcA	Fpo with MPH_2_	HdrED with MPH_2_	Rnf with electrons from MmcA	[31]
*M. thermoacetophila*	MspA	SpqAB with PQQH_2_	Fpo^−^-FrhB complex with MPH_2_, interaction of Fmd and Fdh with Fd_red_^2−^	HdrABC with F_420_H_2_ and HdrED with MPH_2_	HdrABC with F_420_H_2_ and oxidation of acetate	[23]
*M.* *harundinacea* 6Ac	Unknow	Cyt *c*?	HdrABC-FrhB complex with Fd_red_^2−^, CoM-SH and CoB-SH	HdrED with MPH_2_	Fpo^−^ with MPH_2_	This article
